# Accelerated ethanol elimination via the lungs

**DOI:** 10.1038/s41598-020-76233-9

**Published:** 2020-11-12

**Authors:** Jesse M. Klostranec, Diana Vucevic, Adrian P. Crawley, Lashmi Venkatraghavan, Olivia Sobczyk, James Duffin, Kevin Sam, Royce Holmes, Ludwik Fedorko, David J. Mikulis, Joseph A. Fisher

**Affiliations:** 1 Department of Medical Imaging, University of Toronto, Toronto, ON Canada; 2 Division of Neuroradiology, Toronto Western Hospital, University Health Network, Toronto, ON Canada; 3 Division of Diagnostic and Interventional Neuroradiology, Montreal Neurological Institute and Hospital, McGill University Health Centre, Montréal, QC Canada; 4 Department of Materials Science and Engineering, Faculty of Applied Science and Engineering, University of Toronto, Toronto, ON Canada; 5 Department of Anesthesia, University of Toronto, Toronto, ON Canada; 6 Department of Anesthesia and Pain Management, University Health Network, Toronto Western Hospital, Toronto, ON Canada; 7 Thornhill Medical Inc., Toronto, ON Canada; 8 Department of Anesthesia and Pain Management, University Health Network, Toronto General Hospital, University of Toronto, 200 Elizabeth St., Toronto, ON M5C 2E4 Canada

**Keywords:** Physiology, Preclinical research, Translational research

## Abstract

Ethanol poisoning is endemic the world over. Morbidity and mortality depend on blood ethanol levels which in turn depend on the balance between its rates of absorption and clearance. Clearance of ethanol is mostly at a constant rate via enzymatic metabolism. We hypothesized that isocapnic hyperpnea (IH), previously shown to be effective in acceleration of clearance of vapour anesthetics and carbon monoxide, would also accelerate the clearance of ethanol. In this proof-of-concept pilot study, five healthy male subjects were brought to a mildly elevated blood ethanol concentration (~ 0.1%) and ethanol clearance monitored during normal ventilation and IH on different days. IH increased elimination rate of ethanol in proportion to blood levels, increasing the elimination rate more than three-fold. Increased veno-arterial ethanol concentration differences during IH verified the efficacy of ethanol clearance via the lung. These data indicate that IH is a nonpharmacologic means to accelerate the elimination of ethanol by superimposing first order elimination kinetics on underlying zero order liver metabolism. Such kinetics may prove useful in treating acute severe ethanol intoxication.

## Introduction

Ethanol intoxication is endemic world-wide with related morbidity and mortality showing an increase over the last two decades within the United States^1^. At lower blood ethanol concentrations, impaired judgment and coordination make people a risk to themselves and to those around them, but high levels put them at risk for organ damage and death from pulmonary aspiration, respiratory depression, and malignant arrhythmias^2^. Blood levels of ethanol reflect the balance between the rates of gastrointestinal absorption and various routes of elimination. More than 90% of ethanol clearance is via the liver where the rate limiting enzyme alcohol dehydrogenase becomes saturated at relatively low blood concentrations^3–5^. This results in a constant rate of metabolism independent of blood ethanol levels, referred to as Michaelis–Menten zero order kinetics^6^. A critical situation may arise in acute intoxications when continued rapid ethanol absorption is superimposed on already toxic blood levels. Unfortunately, the management options of such life-threatening blood ethanol concentrations are still restricted to supportive measures and attempts at resuscitation if cardiac arrest occurs.

In lieu of some new antidote for ethanol intoxication, an alternative solution is to find a way to accelerate its clearance from the blood. Elimination of ethanol via renal metabolism increases in proportion to blood levels (termed ‘first order kinetics’) involving cytochrome P450, but this accounts for only 2–5% of overall ethanol clearance^7^. It has been noted almost a century ago that the ethanol present in exhaled breath is amenable to a form of elimination proportional to the blood concentration and level of ventilation (minute ventilation)^8^. The authors reported a small case series demonstrating the efficacy of increased levels of minute ventilation in clinical recovery from ethanol. The pharmacokinetics of volatile gas clearance from the blood via the lungs was more recently investigated for other volatile liquid compounds (sevoflurane^9^ and isoflurane^10^) and the gas carbon monoxide (CO)^11, 12^. For the latter, the elimination is proportional to blood levels. Pharmacokinetic considerations would support the same being the case for the lung clearance of all volatile hydrocarbons.

We hypothesized that the combined effects of IH and first order kinetics would significantly accelerate clearance of ethanol from venous blood and thereby from the body despite its high blood solubility and water miscibility. The main aim of the study was to determine to what extent IH can increase the rate of ethanol elimination above baseline liver metabolism. Our general approach was to assess the overall kinetics of blood clearance of ethanol using sequential breathalyzer readings of blood ‘alcohol’ (ethanol) concentrations with and without IH. We also examined the efficacy of IH on lung ethanol elimination by drawing simultaneous arterial and venous blood samples and measuring the veno-arterial ethanol concentration differences during IH. To verify faster ethanol elimination via the lung with IH, we abruptly ceased IH and looked for a “rebound” of arterial ethanol levels.

## Materials and methods

This is a proof of concept pilot study on volunteers in a laboratory setting, with each subject acting as his own control.  We studied 5 healthy male participants, taking no medication and without a history of ethanol abuse (Table 1). Procedures followed approval of the protocols by the research ethics board at the University Health Network and were carried out in accordance with relevant guidelines and regulations, including obtaining informed consent from all subjects. Prior to each experiment the subject had refrained from eating and drinking for 6 h and from alcohol ingestion for 12 h. Study subjects were not allowed to leave the testing area until registering a breathalyzer value less than 0.03% (AlcoHAWK, Quest Products, Pleasant Prairie, WI). Each subject attended two sessions on separate days. The order of the protocols was randomized by blindly choosing a folded paper from an envelope with an equal number of “Baseline” and “IH” tabs. All subjects agreed not to drive a car for the remainder of the study day, and arranged to be accompanied home after the experiment.

For each session, end-tidal PCO_2_ (PetCO_2_), minute ventilation (MOVES^R^, Thornhill Medical Inc., Toronto, Canada), and breathalyzer readings were taken at baseline. The participant then ingested 0.5 g ethanol/kg of body weight over 5 min. The ethanol was constituted from 40%/volume vodka mixed in 500 ml of mineral water. For the pharmacokinetic studies, breathalyzer measurements were initiated 20 min after ingestion and continued at 5 min intervals for approximately 3 h. In the Control protocol, the subject was seated and continued to breathe room air for 3 h.

### Isocapnic hyperpnea (IH) protocol

The sequential gas delivery (SGD) theory and apparatus used for implementing IH was first described by Sommer et al*.* about 20 years ago^13^. The method has more recently been reviewed with an expanded explanation^14, 15^. In brief, the core concept for SGD is that exhaled gas is considered to have been in equilibrium with alveolar capillary blood. Thus, absent a partial pressure gradient, exhaled gas does not participate in CO_2_ exchange with the blood. Let us call this previously exhaled gas ‘neutral gas’. Thus, if a fixed volume of fresh gas is available to be inhaled, followed by an unlimited volume of neutral gas, the former is inhaled into the alveoli and is the sole determinant of the CO_2_ exchange regardless of how much additional neutral gas is inhaled. In a system where the fresh gas comes from a constant flow, as long as that flow is less than the subject’s minute ventilation, the fresh gas flow determines the alveolar ventilation (i.e., CO_2_ elimination). This holds true regardless of the sizes (tidal volume) or consistency of the breaths or the minute ventilation. But, as ethanol diffuses into all the gas in the alveoli—the fresh gas plus the neutral gas—the ethanol elimination will be determined by the total minute ventilation. Hence, SGD maintains isocapnia while accelerating the elimination of all gases and dissolved volatile molecules not present in inspired gas.

**Figure 1:**
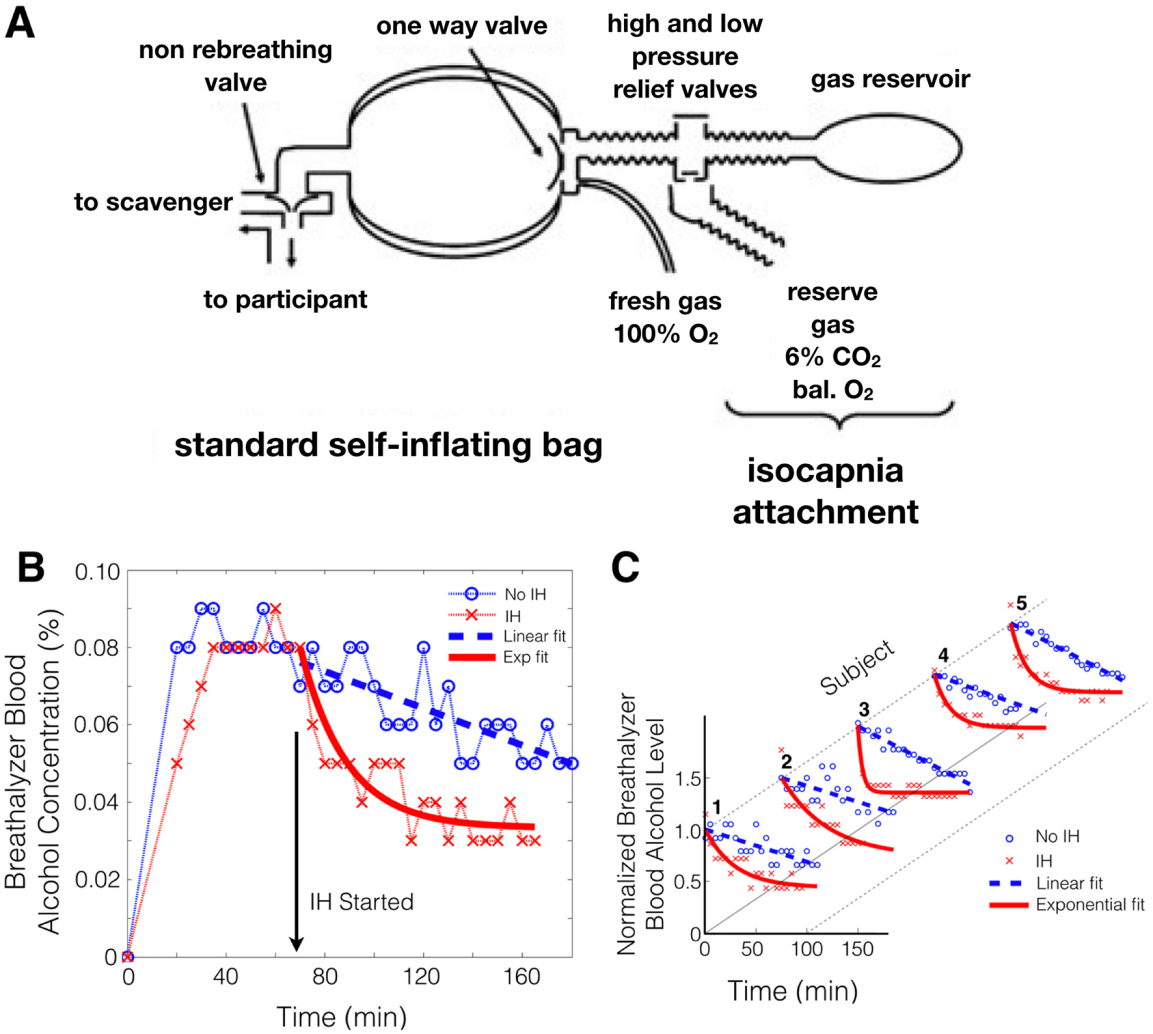
Effect of isocapnic hyperpnea (IH) on ethanol elimination based on breathalyzer blood ethanol concentration measurements. (**A**) Schematic of the ClearMate^TM^ passive non- rebreathing, hyperoxic, IH circuit. (From Ref.^15^, Copyright (2011) John Wiley and Sons, Inc. Reprinted with permission from John Wiley and Sons, Inc.) (**B**) Breathalyzer measurements in subject 1 without and with IH started at the 70 min mark after alcohol ingestion. Linear, zero order elimination kinetics are demonstrated without IH (blue dashed line) while exponential first order elimination kinetics are demonstrated with IH (red line). (**C**) Normalized breathalyzer measures versus time for 5 male subjects demonstrating consistent zero order elimination kinetics without IH (dashed blue line) compared with first order elimination kinetics with IH (red line).

The ClearMate^TM^ (Thornhill Medical Inc, Toronto, ON, Canada) implements SGD by providing for alveolar ventilation via an oxygen flow into the reservoir of a self-inflating bag^16^. Once this is inhaled, the balance of inhaled gas consists of “neutral gas” composed of 5.5% CO_2_ balance O_2_, delivered via a demand regulator (Fig. 1A).

Breathalyzer readings were monitored after ingestion. Once test-to-test breathalyzer readings stopped increasing to indicate a slowing rate of gastric absorption (approximately 60 min post ingestion), the subject held an occlusive anesthesia mask to his face and breathed via the IH circuit (Fig. 1A). Subjects found it more comfortable and easier to maintain a seal by holding the masks themselves rather than using head straps. Subjects voluntarily increased minute ventilation from approximately 4–5 L/min when at rest to 30 L/min when performing IH. The net inspired fraction of O_2_ (FiO_2_) was about 0.95; the O_2_ flow (i.e. the fresh gas flow) was adjusted by the investigators to maintain PetCO_2_ within the subject’s comfort range. For example, the O_2_ flow was increased if the subject signaled that he preferred a reduction in his PCO_2_. In addition, the O_2_ flow was reduced slightly to raise the PCO_2_ and provide a mild respiratory drive to help the subject sustain hyperpnea. All adjustments were made with the cooperation of the subject. IH was interrupted every 5 min to make measurements with the breathalyzer. Subjects were instructed to cease IH and breathe room air normally at rest. After 30 s of quiet breathing, a series of three measurements was made. Subjects were instructed to take a normal inspiration and breathe out slowly to residual volume into the breathalyzer; this was to optimize the equilibration of alveolar gas with the blood in the capillaries prior to measurement. The first two measurements were discarded to allow at least 90 s for equilibration of the pulmonary gases with arterial blood. Breathalyzer measurements were continued periodically for 60–90 min until no significant change in readings was observed in three consecutive measurements.

### Rebound of breathalyzer readings following IH

Ethanol ingestion and the initiation of IH were performed as described above. Again, IH was interrupted every 5 min to make three breathalyzer measurements, with the first two discarded. After 30 min, IH was not re-commenced while breathalyzer measurements continued every 5 min. A second period of IH was then performed for 20 min, with breathalyzer measurements continuing every 5 min.

### Arterial and venous blood samples with IH

Ethanol was ingested as described above. An intravenous catheter was placed in the right antecubital fossa and an arterial catheter was placed in the left radial artery using local anesthetic and sterile technique. Baseline and intermittent arterial and venous blood samples were drawn. An occlusive oxygen-type mask was fixed to the face using skin tape (Tegaderm, 3M St. Paul, MN, USA) to provide an interface for the IH and to sample end-tidal gases. Arterial and venous blood samples were collected for laboratory assessment of blood ethanol levels every 5 min. Following the experiments, the catheters were removed and participants continued IH until breathalyzer readings were < 0.03%.

### Data analysis

Line and curve fitting was performed using a least-squares method in Matlab (linearizing exponential data by taking the natural logarithm), with the results given in Table 1.

**Table 1:** Study participant information.

Subject	Age	Weight (kg)	non-IH BrAC			IH BrAC			
			Linear slope, *m* (%/min)	y-intercept, *b* (%)	t½ (min)	*A*_0_	α	*C*_0_	t½ (min)
1	34	72	− 0.00024	0.093	166.7	0.039	− 0.034	0.030	63.8
2	68	74	− 0.00028	0.110	142.9	0.042	− 0.022	0.013	48.6
3	72	90	− 0.00049	0.170	102.0	0.060	− 0.230	0.033	6.6
4	27	122.5	− 0.00028	0.075	178.6	0.051	− 0.063	0.048	56.1
5	20	70	− 0.00042	0.087	107.1	0.080	− 0.063	0.039	21.6

*IH* isocapnic hyperventilation, *BrAC* breathalyzer blood ethanol concentration; linear decay assumes form *y* = *mx* + *b*; exponential decay assumes the form y = *A*_0_exp(α*t*) + *C*_0_. Confidence intervals are calculated using two tailed *t*-tests.

## Results

All subjects completed both control and test phases of the study. All subjects found the prolonged hyperpnea tedious but not uncomfortable or requiring great effort. Breathalyzer readings rose rapidly as a result of ethanol absorption from the upper gastrointestinal tract and reached a plateau at about 50–60 min. Thereafter, a slow linear decline in readings occurred during normal respiration (dashed blue lines in Fig. 1B,C; Table 1). When each subject performed IH from a plateau ethanol level, the breathalyzer readings declined following an exponential decay pattern reflecting first order kinetics (red lines in Fig. 1B,C; Table 1). Despite the mild initial ethanol intoxication under these test conditions, the mean half-life of elimination, *t*½_,_ with IH resulted in a clearance rate three times greater than that of baseline, with 95% confidence intervals of 139 ± 38 min and 39 ± 27 min without and with IH, respectively (Table 1).

**Figure 2:**
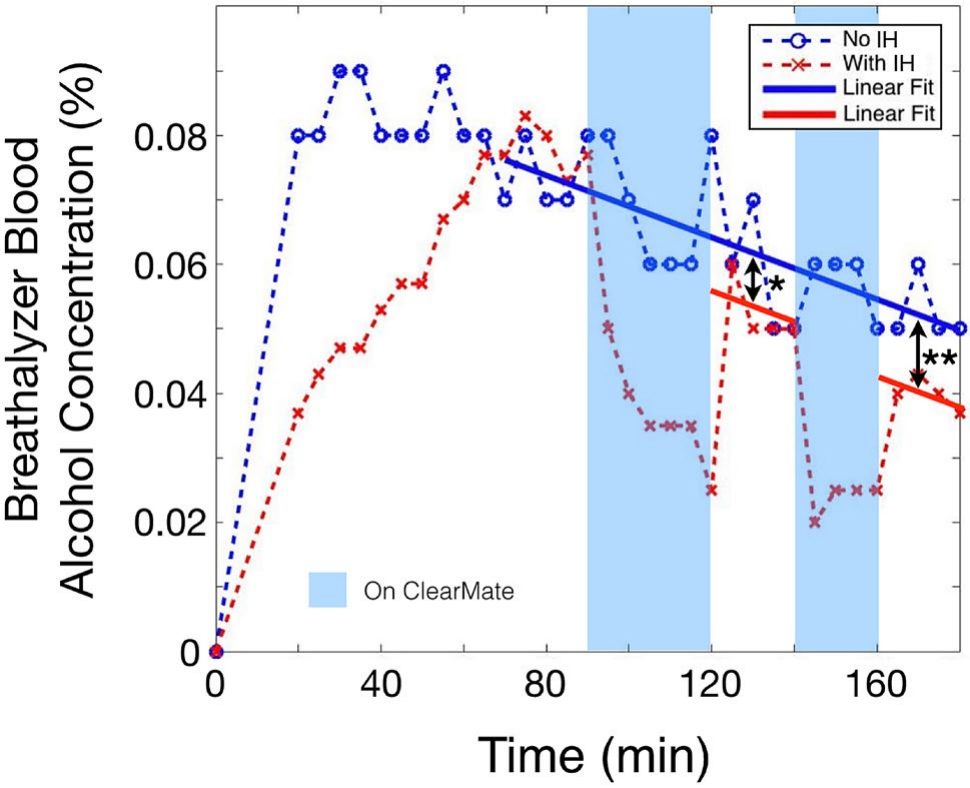
“Rebound” effect with intermittent IH. Breathalyzer measurements of subject 1 with two periods of intermittent IH demonstrate first order ethanol elimination kinetics while IH is performed, with a “rebound” increase in blood ethanol concentration when IH is ceased, followed by return to zero order elimination kinetics (red lines). However, the “rebound” occurs to a level below that expected if no IH had been performed, as demonstrated by offsets in the linear elimination kinetics after the first (*) and second (**) IH period; the offset increasing with the total duration of IH (black double arrows).

**Figure 3:**
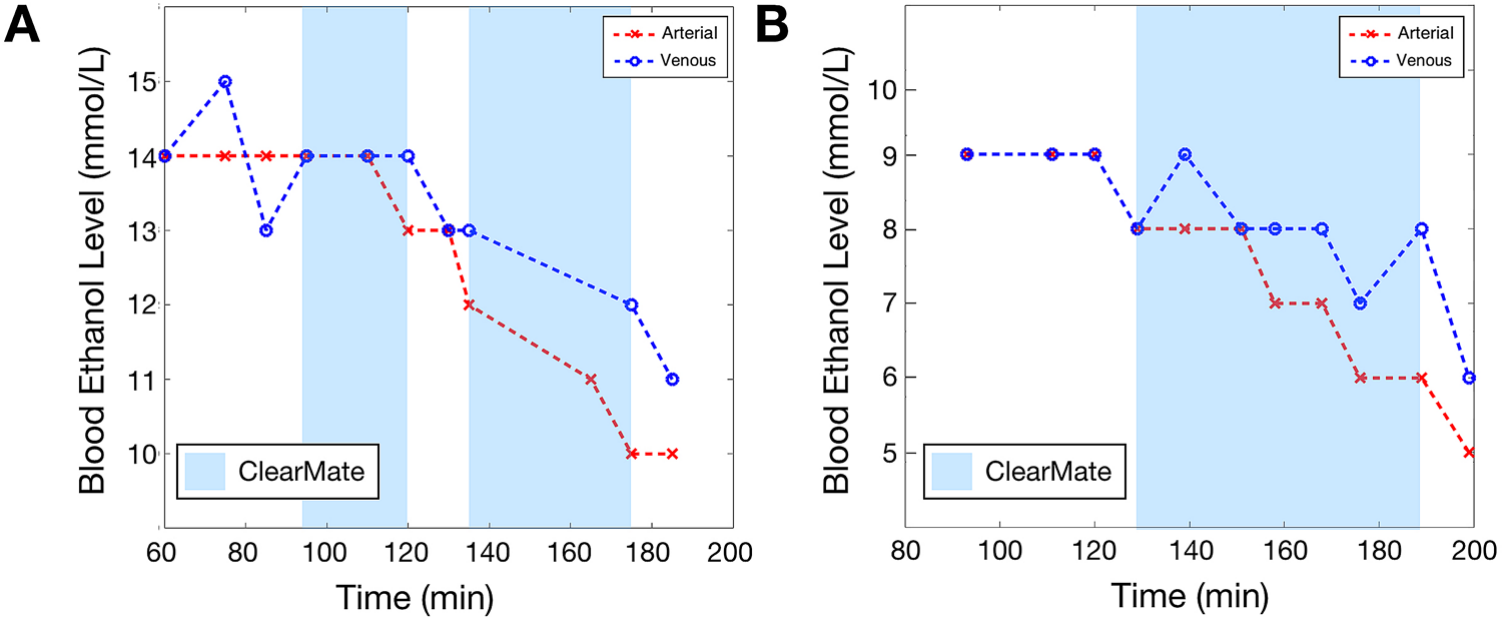
Effect of IH on blood ethanol concentration. (**A**) Arterial and venous blood ethanol concentration measurements in subject 1 with two intermittent periods of IH performed. Clear establishment of a veno-arterial gradient that increases in magnitude with duration of IH is demonstrated. Similar results were observed in subject 2 (**B**).

A small degree of rebound of arterial ethanol concentration with abrupt cessation of IH was observed in two participants (illustrated in Fig. 2 for subject 1), indicating the effectiveness of IH at lowering mixed venous ethanol concentrations during transit in the lungs. We observed that zero order clearance (i.e., a constant rate) resumed after IH was stopped (* and ** in Fig. 2), providing a contrast to the enhanced pulmonary ethanol elimination during IH. Under IH, veno-arterial blood ethanol gradients demonstrated up to a 20% difference (Fig. 3A,B), further indicating the efficacy of lung clearance of ethanol with the increase in minute ventilation.

## Discussion

### IH-enhanced ethanol clearance

The main finding of this study is that IH induced an exponential clearance of ethanol with a short *t*½ increasing the absolute rate of clearance by at least a factor of 3, even at the low initial intoxication levels of this experiment. At greater levels of intoxication, the t½ would have been the same but the absolute rate of elimination would have been proportionally greater. This accelerated clearance is induced by IH via the combined effect of increased gas washout from the lung and the effect of first order kinetics, which implies that clearance is proportional to blood ethanol concentration.

**Figure 4:**
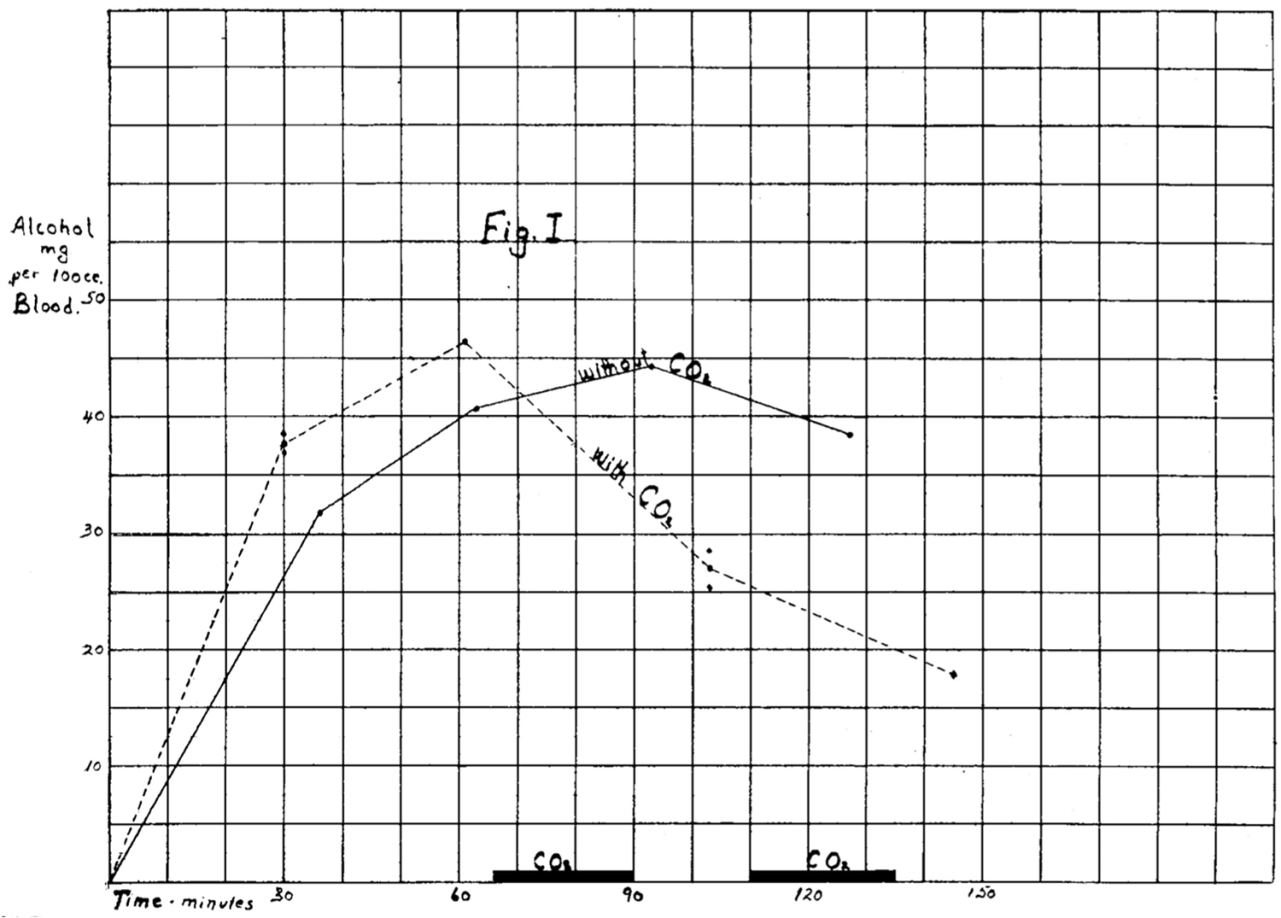
Blood alcohol concentration after ingestion in a test subject with and without administration of CO_2_. “The solid line representing the blood alcohol concentration on the first day when no carbon dioxide was given, and the broken line the concentration on the second day when carbon dioxide was administered. The shaded area at the bottom of the chart shows the time during which carbon dioxide was given on this day.” (From Ref.^8^, Copyright (1924) Massachusetts Medical Society. Reprinted with permission from Massachusetts Medical Society.).

The effect of IH on the rate of elimination of CO^11^ and volatile anesthetics^9^ from the blood has been reported in just the last two decades. However, almost a century ago Hunter and Mudd reported an increase in the rate of reduction of blood ethanol levels in a single subject following CO_2_-stimulated hyperpnea^8^. They did not appreciate the first order kinetics of the ethanol elimination from the few measurements they performed; however, their data (Fig. 4) otherwise appears remarkably similar to our Fig. 1. Our study extends their observations by the first demonstration of (a) the superimposition of an exponential elimination pathway on the saturated enzymatic ethanol metabolism; (b) the effect size via the veno-arterial ethanol concentration gradient; and (c) the use of CO_2_ in the form of IH to support (rather than drive) the hyperpnea.

The reason hyperpnea as a form of treatment for ethanol intoxication has been neglected over the last century may be that the effect size of IH on ethanol clearance is counterintuitive, and thus, unexpected. Unlike CO, which is confined to the blood compartment, ethanol is highly water soluble and thus distributes to a much greater extent into interstitial and intracellular fluid. This aspect may have suggested to some that it would have a reduced tendency to cross into the gas phase in the alveoli. Our study showed that nevertheless, in practice, IH markedly increases ethanol elimination. IH is effective because, rather than the blood/gas partition coefficient, it is the vapor pressure of the gas that determines the volume percent concentration achievable in the alveolar gas phase which also determines mass transfer into the gas phase and subsequent elimination from the lungs^8^. The vapor pressure of ethanol (16 kPa) is of the same order of magnitude as that of sevoflurane (40 kPa), for which IH markedly shortens the time of recovery of consciousness in humans^10^. Hunter and Mudd also noted that methanol, which has an even higher vapor pressure than ethanol (30.7 kPa at 37 °C), should also have a greater elimination rate with IH^8^. Should preclinical studies confirm such efficacy, the IH approach would fit well with the recent introduction of alcohol dehydrogenase inhibitors as a non-invasive treatment for methanol intoxication.

Infusing CO_2_ into an open mask provides a variable stimulus, the effect of which depends on the state of consciousness of the patient, patient tolerance of hypercapnia, the presence of other drugs in the blood, and the chemoreceptor-induced ventilatory response to the CO_2_. The enabling technology for IH, which uses a pneumatic mechanism (no electronics or computers) to provide CO_2_ to inspired gas in the exact proportion to replace exhaled CO_2_ for any minute ventilation and breathing pattern, and thereby maintain constant arterial PCO_2_, has been known for just the past 20 years^13^. The hyperoxia-induced increases in minute ventilation and maintenance of isocapnia occur without distress or even conscious ventilatory effort on behalf of the patient^14, 17^. This functionality, and the ready availability of regulatory approved technology, make IH potentially rapidly adoptable to clinical management of problematic ethanol intoxications.

The prospect of induced passive IH as a therapeutic intervention has a number of considerations. From the pharmacokinetic perspective, it is especially important in cases of severe intoxication when rising ethanol levels from continued gut absorption can be life-threatening. In these cases there are no other known prophylactic or antidote mitigations. An important clinical implication of first order kinetics is that the highest clearance efficiency and the greatest absolute rates of ethanol elimination would occur when most needed: at the highest ethanol blood levels and toxicity, when other available supportive measures are least effective. The wide veno-arterial ethanol gradient on initiation of IH in our study indicates that it could provide almost immediate reduction in arterial ethanol levels—and thus in highly perfused organs such as the brain and heart—that can be maintained with IH (see principle in Lemburg et al.^18^), which itself can be sustained due to the maintenance of normocapnia at all minute ventilations. To the extent that ethanol or any other volatile toxin is cleared via the lung, the arterial concentration is less than that of the tissues. The greater the organ perfusion, and the lower the arterial ethanol concentration (i.e., the greater the ventilation), the lower the net tissue ethanol concentration. If ventilation is abruptly halted, the arterial ethanol concentrations rises toward the venous levels, which are in equilibrium with tissue levels. In referring to this as ‘rebound’, we must keep in mind that neither the arterial nor the tissue concentration rise above the mean body ethanol concentration.

From the practical bedside perspective, applying IH to heavily intoxicated patients who require critical care that includes endotracheal intubation, would simply requires manual ventilation with a self-inflating bag. With a t½ of about 40 min seen in this study, it can be anticipated that much less time would be required to reduce blood levels below a lethal range.

This study was performed using only 5 human subjects. The consistency of their pharmacokinetic data provided confidence to conclude that reproducible ethanol clearance features of IH include (i) first order kinetics and (ii) an effect size on clearance sufficient to distinguish it from liver metabolism. The efficacy and large effect size was further confirmed by demonstrating a 20% difference in venous and arterial ethanol concentrations during IH in two subjects. Doubling or tripling the cohort size would still be inadequate to provide population metrics and would not have increased confidence in the results. Adding female subjects can be expected to demonstrate different elimination and “rebound” kinetics resulting from sex-related differences in body ratios of water to fat^3^. Other populations not included but of potential clinical interest in follow-up studies are those with compromised or limited hepatic and/or renal function.

We used breathalyzer ethanol measures as a surrogate for blood ethanol levels for pharmacokinetic measures. Although measurements obtained using a breathalyzer have been reported to accurately reflect blood ethanol levels^17^, other investigations into the dynamics of ethanol removal from the airways have suggested underestimations of blood ethanol levels by as much as 20%^18, 19^. Our aim was to study the kinetics of elimination rather than identify exact blood ethanol levels. To minimize the difference between breathalyzer readings at baseline and during IH, the breathalyzer measurements were taken approximately 90 s following cessation of IH and included three prolonged 20–30 s exhalations supporting the re-establishment of the baseline arterial-venous equilibrium, and thus avoiding repeatedly placing arterial and venous catheters in all of our subjects for all phases of these studies.

## Conclusion

We have presented the first data in humans to suggest IH converts overall ethanol clearance from a rate that is constant (zero order kinetics) to one that is proportional to blood concentration (first order kinetics). The therapeutic promise of enhanced first order kinetics with IH is that the elimination rate of ethanol becomes proportional to the blood level and minute ventilation; the greater the minute ventilation, the faster is the clearance of ethanol from the blood by the lungs and the greater the reduction in arterial, and thus highly perfused organ, ethanol concentration. The veno-arterial ethanol gradient and the rebound of ethanol levels on cessation of IH confirm a substantial effect size of IH in clearing ethanol via the lungs. The path to potential clinical application is shortened by the IH technology having received regulatory approval for treatment of CO poisoning^20^. We suggest follow-up studies to confirm the effectiveness of IH on ethanol elimination in various clinical conditions and proceeding to clinical trials.
